# Intracerebral Proinflammatory Cytokine Increase in Surgically Evacuated Intracerebral Hemorrhage: A Microdialysis Study

**DOI:** 10.1007/s12028-021-01389-9

**Published:** 2021-11-30

**Authors:** Lovisa Tobieson, Anna Gard, Karsten Ruscher, Niklas Marklund

**Affiliations:** 1 Departments of Neurosurgery in Linköping and Biomedical and Clinical Sciences, Linköping University, Linköping, Sweden; 2 Department of Clinical Sciences Lund, Neurosurgery, Lund University, Lund, Sweden; 3 Department of Clinical Sciences Lund, Neurosurgery, Lund University, Skåne University Hopsital, Lund, Sweden

**Keywords:** Brain injury, Cytokine, Inflammation mediators, Intracranial hemorrhage, Microdialysis, Stroke

## Abstract

**Background:**

Treatment options for spontaneous intracerebral hemorrhage (ICH) are limited. A possible inflammatory response in the brain tissue surrounding an ICH may exacerbate the initial injury and could be a target for treatment of subsequent secondary brain injury. The study objective was to compare levels of inflammatory mediators in the interstitial fluid of the perihemorrhagic zone (PHZ) and in seemingly normal cortex (SNX) in the acute phase after surgical evacuation of ICH, with the hypothesis being that a difference could be demonstrated between the PHZ and the SNX.

**Methods:**

In this observational study, ten patients needing surgical evacuation of supratentorial ICH received two cerebral microdialysis catheters: one in the PHZ and one in the SNX that is remote from the ICH. The microdialysate was analyzed for energy metabolites (including lactate pyruvate ratio and glucose) and for inflammatory mediators by using a multiplex immunoassay of 27 cytokines and chemokines at 6–10 h, 20–26 h, and 44–50 h after surgery.

**Results:**

A metabolic crisis, indicated by altered energy metabolic markers, that persisted throughout the observation period was observed in the PHZ when compared with the SNX. Proinflammatory cytokines interleukin (IL) 8, tumor necrosis factor α, IL-2, IL-1β, IL-6 and interferon γ, anti-inflammatory cytokine IL-13, IL-4, and vascular endothelial growth factor A were significantly higher in PHZ compared with SNX and were most prominent at 20–26 h following ICH evacuation.

**Conclusions:**

Higher levels of both proinflammatory and anti-inflammatory cytokines in the perihemorrhagic brain tissue implies a complex role for inflammatory mediators in the secondary injury cascades following ICH surgery, suggesting a need for targeted pharmacological interventions.

**Supplementary Information:**

The online version contains supplementary material available at 10.1007/s12028-021-01389-9.

## Introduction

Spontaneous supratentorial intracerebral hemorrhage (ICH) represents the most severe form of stroke [1]. Current medical treatment options are limited, aimed at preventing ICH expansion [2, 3], and the role of surgery in improving outcome remains controversial [4–7].

The ICH causes an initial primary injury to the brain by direct tissue destruction and a possibly increased intracranial pressure (ICP). Subsequently, the blood break-down products and/or the increased ICP cause an additional secondary brain injury by initiating reactive cellular, metabolic, and neurotoxic cascades. The tissue surrounding an ICH, the perihemorrhagic zone (PHZ), shows an acute hypometabolic and hypoperfusion state [8–10], including mitochondrial dysfunction and metabolic failure [11, 12], and this region may be particularly vulnerable to secondary injury. Experimental ICH studies demonstrate an inflammatory response that includes the rapid activation of microglia [13–16], followed by the infiltration of inflammatory cells over hours and days [17], which may cause additional tissue damage. Improved neurologic outcome is achieved by attenuating the inflammatory process in experimental ICH [18–21]; however, this has failed to translate into pharmacological treatment options for patients with ICH [22]. Early attempts to attenuate the ICH-induced inflammation by using glucocorticoid administration resulted in impaired clinical outcome [23], emphasizing the complex contribution of inflammation to outcome.

Cerebral microdialysis (MD) is a useful clinical tool used to monitor the microenvironment of the brain tissue in the neurocritical care patient [24–26]. Catheters with a larger pore size enable sampling of inflammatory mediators from the interstitial fluid of brain tissue in humans [27] and in animal models [28], and the cerebral interstitial cytokine expression has been explored in patients with subarachnoid hemorrhage (SAH) and traumatic brain injury (TBI) [27, 29–34], but not in patients with ICH. Several studies, however, have explored the plasma concentration of proinflammatory mediators such as interleukin (IL) 6 [35–38] and tumor necrosis factor (TNF) α [37, 38] in patients with ICH, indicating that inflammation plays a role in secondary brain injury following ICH.

In this study, we sampled interstitial fluid by using paired MD catheters, one in the PHZ and one in seemingly normal cortex (SNX), to investigate changes in inflammatory mediators in the acute phase after the surgical evacuation of ICH. We hypothesized a difference in cytokine and chemokine levels in the PHZ compared with those in the SNX.

## Materials and Methods

### Ethics

The study protocol was approved by the regional ethical committee in Linköping, Sweden (decision number 2014/236-31) and was conducted in accordance with relevant guidelines and regulations. Informed consent was obtained from the patient’s closest relative.

### Patients

Adults requiring acute ICH evacuation via open craniotomy at the Department of Neurosurgery, University Hospital, Linköping, Sweden in 2016–2018 were prospectively recruited to this observational cohort study. Severe coagulation disorders and a known source of bleeding, such as an aneurysm or an arteriovenous malformation, were exclusion criteria. ICP monitoring was achieved by using standard protocols adopted by our department [39]. One MD catheter (CMA-71 Brain Catheter; M-Dialysis, Solna, Sweden) was inserted via the craniotomy into the PHZ within 1 cm of the evacuated ICH, and the other catheter was inserted in the SNX, as previously described [39]. A postoperative computed tomography (CT) scan was performed to verify MD catheter placement [40, 41]. Systemic inflammatory parameters in the blood were analyzed daily. Patients were treated according to a standardized neurocritical care protocol to avoid secondary insults [39]. Continuous electroencephalogram was initiated, when indicated, to rule out nonconvulsive seizure activity (*n* = 1). Three patients were included in a previous publication from our group [39]. Patient records were investigated at 12 months to determine clinical outcome determined with the modified Rankin Scale (mRS).

### Microdialysis

Microdialysis catheters with 10-mm membrane length and a molecular weight cutoff of 100 kDa (CMA-71) were perfused with 5% human albumin in a water solution (Albunorm, 50 g/l; Octapharma AB, Stockholm, Sweden), at a rate of 0.3 µl/min using the CMA 106 perfusion pump (M-Dialysis AB) [27]. The first 2 h of sampling were discarded according to consensus praxis [26]. Samples were collected every 2 h for routine analysis of low-molecular weight metabolites (glucose, lactate, pyruvate, glycerol, and glutamate) [26, 42]. The remaining sample (30 µl/vial) was stored at − 86 °C until further analysis was completed.

### Analytical Methods

#### Metabolite Analysis

Interstitial levels of glucose, lactate, pyruvate, glycerol and glutamate in the MD samples were analyzed bedside using an ISCUS Flex analyzer (M-Dialysis AB, Solna, Sweden), as previously described in detail [39].

#### Multiplex Immunoassay

MD samples from three postoperative time points; early (4–10 h), intermediate (20–26 h), and late (44–50 h after surgery), were analyzed for inflammatory mediators using the Meso Scale Discovery (Rockville, MD) MULTI-SPOT Assay System V-PLEX Human Proinflammatory Panel 1, Cytokine Panel 1, and Chemokine Panel 1(cat #K15210D). The proteins were detected by immunoassays, using electrochemiluminescent labels, providing a quantitative measure of protein concentration.

To achieve a sufficient sample volume (25 µl), 3 MD vials were pooled, resulting in a 6-h time resolution. The pooled samples were diluted to one fifth of the original concentration. All samples were analyzed with the same protocol by blinded investigators.

#### Antibody Solution

V-PLEX Proinflammatory Panel 1: 67 μl of SULFO-TAG Antihuman interferon (IFN) γ, IL-1β, IL-2, IL-4, IL-6, IL-8, IL-10, IL-13, and TNF-α were added to 2400 μl of diluent.

V-PLEX Cytokine Panel 1: 67 μl of SULFO-TAG Antihuman IL-1α, IL-5, IL-7, IL-12/IL-23p40, IL-15, IL-16, IL-17A, TNF-β, and vascular endothelial growth factor A (VEGF-A) were added to 2400 μl of diluent.

V-PLEX Chemokine Panel 1: 67 μl of SULFO-TAG Antihuman Eotaxin, macrophage inflammatory protein (MIP) 1β, Eotaxin-3, thymus and activation regulated chemokine, IP-10, MIP-1α, monocyte chemoattractant protein 1, macrophage derived chemokine, and monocyte chemoattractant protein 4 were added to 2400 μl of diluent.

#### Assay Protocol

After blocking with blocker H, shaking for one hour, plates were washed three times with 150 μl phosphate buffered saline (PBS + 0.05% Tween 20; wash cycle). Sample or calibrator (25 μl) was added and plates were sealed and incubated at room temperature with shaking for 2 h, followed by three wash cycles. Antibody solution (25 μl) was then added to each well, followed by incubation at room temperature with shaking for two hours, and subsequently three wash cycles. Finally, 2X Read Buffer T (150 μl) was added, and the plates were analyzed with a MESO QuickPlex SQ 120 instrument.

### Statistical Analyses

Statistical analyses were performed in SIMCA 17.0.0 (Umetrics, Sweden) or in IBM SPSS 27.0 (IBM, Kista, Sweden). Data distribution was assessed using Shapiro-Wilks’ normality test. Low-molecular weight metabolite data were analyzed using linear mixed model (MML) method using patient number as subject level and catheter location as fixed effect. Univariate analysis was performed using paired Wilcoxon signed-rank test for nonnormally distributed data. Values below lower limit of detection (LLOD) of the assay were substituted with the exact value given for LLOD by the manufacturer. Multivariate analysis (MVA) was performed by overviewing the data using principal component analysis (PCA) and thereafter fitting an orthogonal projection to latent structures discriminant analysis (OPLS-DA) model to the data. Critical outliers were investigated with Hotelling’s T2 in the PCA. Moderate outliers were investigated using distance to model X (DModX) [43]. Model validity was investigated with a cross-validated ANOVA (CV-ANOVA) and a *p* value < 0.05 considered significant. Scaling to unit variance and mean centering was employed. Variables with a │p(corr)│ > 0.4 and variable influence on projection (VIP) > 1 were considered significant [43]. Hierarchical cluster analysis was performed using Ward’s method. Network analysis was performed using the STRING database.

## Results

**Fig. 1:**
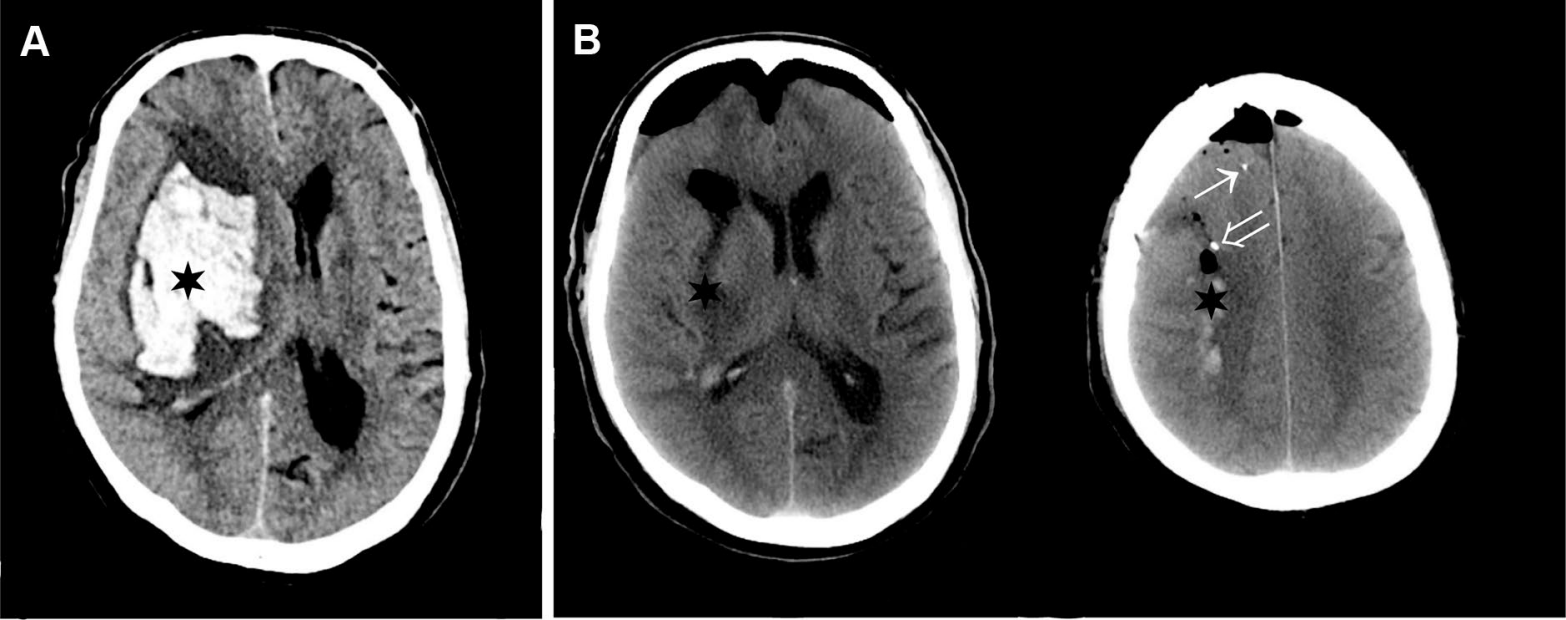
Preoperative and postoperative CT scan. **a,** Preoperative CT scan of a 68-year-old man presenting with an intracerebral hemorrhage (ICH) (black star) in the right basal ganglia. **b,** Following surgical evacuation of the ICH, a postoperative CT scan shows the hematoma cavity (black star) and the tip of the microdialysis (MD) catheters placed in the perihemorragic zone (PHZ) open arrow and seemingly normal cortex (SNX) (closed arrow), respectively. CT= computed tomography

Ten patients (eight men, two women; Table 1) underwent surgery with evacuation of ICH and placement of dual MD catheters (Fig. 1a, b). None of these patients had intraventricular hemorrhage, and an external ventricular drain (EVD) was not inserted. ICP monitoring was initiated in three patients using a parenchymal ICP monitor. Median age was 64 (51–71) years; median time to surgery from ICH onset was 27.8 (6–82) hours and median MD-sampling time was 95 (50–148) h. Mean distance from MD catheter to evacuated ICH was 6 (± 5.0) mm for the PHZ catheter and 24.5 (± 7.6) mm for the SNX catheter. Mean volume of ICH was 77 (± 25.8) ml (Table 1). The PHZ catheter of patient 10 was not placed according to protocol (> 10 mm from ICH), however, on CT control, it was located in tissue affected by the ICH and was included in the analysis. The SNX catheter of patient 7 was placed in tissue which subsequently developed an ischemic infarction thus not representing normal cortex, and the data from this MD catheter was excluded from further analysis. White blood cell count was 9.6 (± 1.9), 10.1 (± 1.6), and 9.2 (± 2.5) × 10^9^ at 6–10 h, 20–26 h, and 40–46 h, respectively. C-reactive protein was 25.7 (± 28), 99.3 (± 72) and 131.4 (± 78) mg/l at 6–10 h, 20–26 h and 40–46 h, respectively. Functional outcome at 12-month follow-up is presented in Table 1.

### Metabolites

**Fig. 2:**
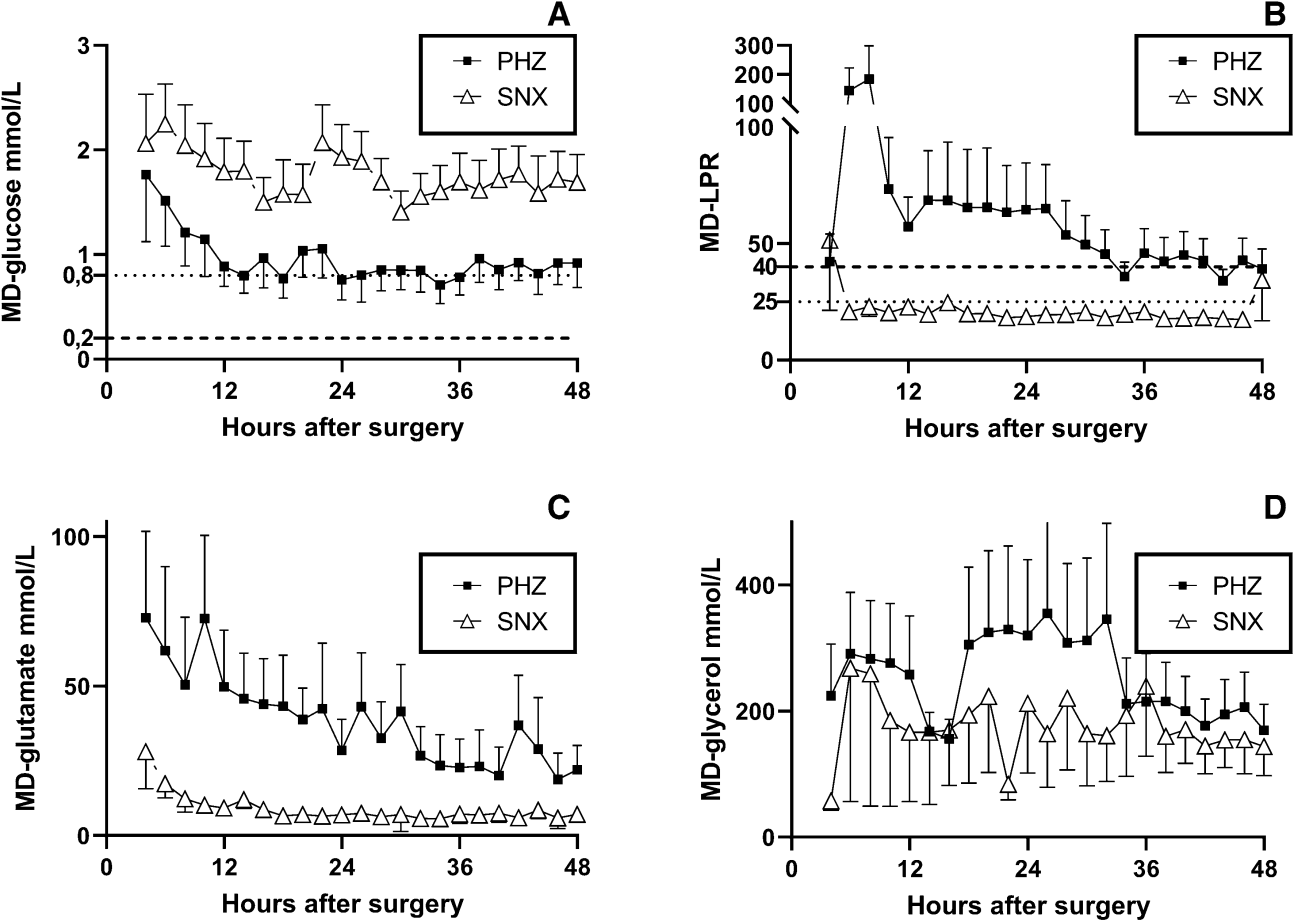
Metabolic crisis in the PHZ. **a,** Microdialysis (MD) glucose levels were significantly lower in the perihemorrhagic zone (PHZ) when compared with those in seemingly normal cortex (SNX) (*p* < 0.05), although were consistently above critical (0.2 mmol/L) and warning levels (0.8 mmol/L) in both locations [26]. **b,** The LPR, however, was pathologically elevated in the PHZ, indicating a metabolic crisis in the brain tissue. In contrast, the SNX LPR normalized within the first hours after surgery, and thereafter remained within the normal range. **c** MD-glutamate levels decreased with time in the PHZ but were significantly higher than in the SNX (*p* < 0.05) during the initial 48 h. **d,** In addition, MD-glycerol levels were persistently higher in the PHZ when compared with the SNX (*p* < 0.05). Data are presented as mean and standard error of the mean (SEM) for clarity. LPR= lactate pyruvate ratio

MD-glucose levels were significantly lower in PHZ compared with SNX (Fig. 2a; *p* < 0.05), however, consistently above the 0.2 mmol/l critical level [26] in both locations, suggesting adequate substrate delivery. Nevertheless, the lactate-pyruvate ratio (LPR) was significantly higher in PHZ than in SNX (Fig. 2b; *p* < 0.05), indicative of a metabolic crisis in the PHZ [39], and was particularly evident early following surgery but persisting beyond 48 h. MD-glutamate and MD-glycerol levels were also significantly higher in PHZ than in SNX (Fig. 2c, d; *p* < 0.05).

### Cytokines and Chemokines

All inflammatory mediators were in detection range although their expression was highly variable with a fourfold dynamic range. TNF-β was recovered in < 2% of the samples and was excluded from analysis. In total, 1,512 multiplex analysis results were obtained.

### Univariate Analysis

**Fig. 3:**
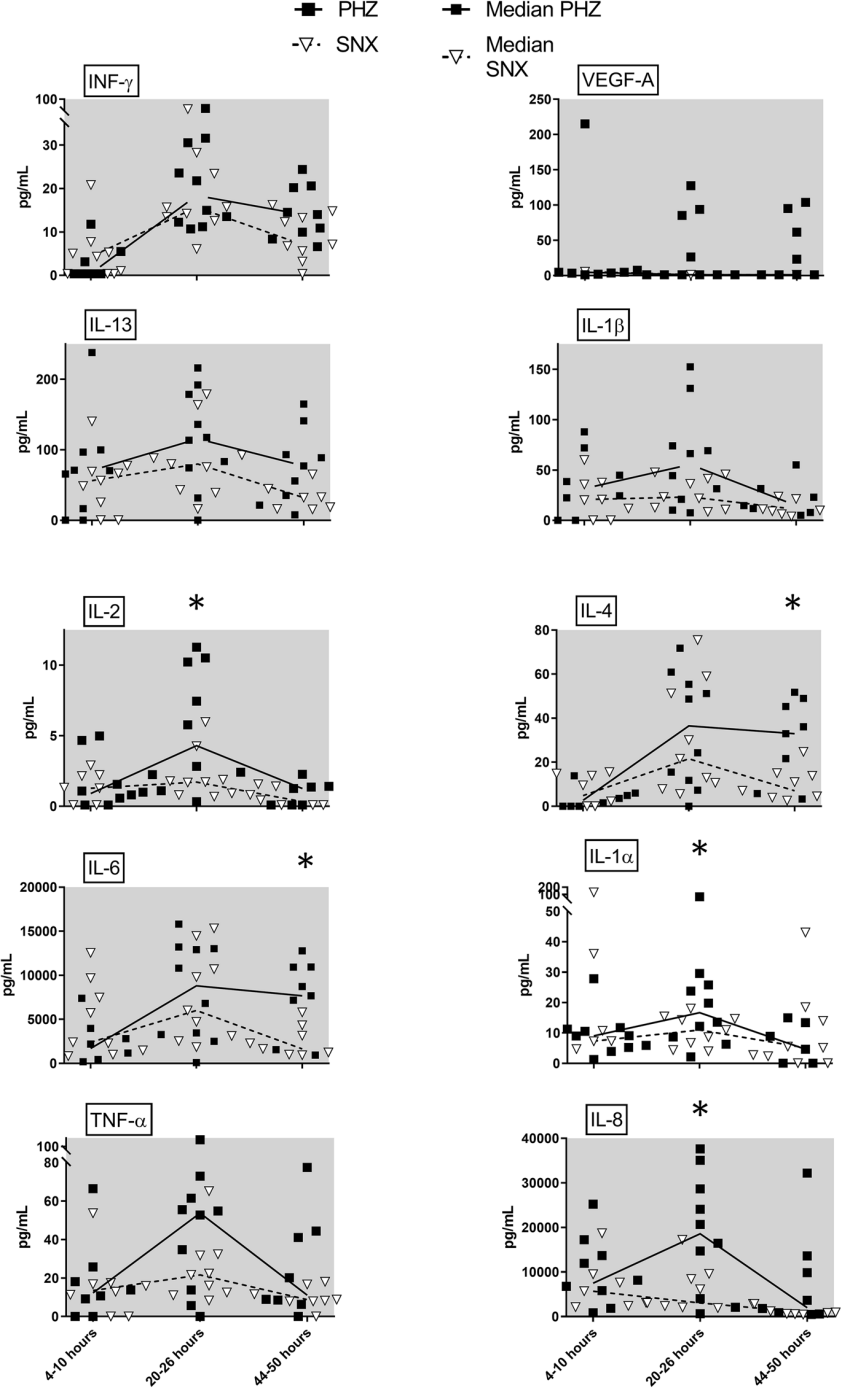
Univariate analysis of cytokines. Significantly higher levels of IL-2 and IL-8 were seen in the perihemorrhagic zone (PHZ) compared with seemingly normal cortex (SNX) at 20–26 h after surgery. At 44–50 h after surgery, there was a significantly higher expression of IL-4, which is considered an anti-inflammatory cytokine, and IL-6, which is considered a proinflammatory cytokine, in the PHZ compared with the SNX. Several of the cytokines contributing to group separation in the supervised OPLS-DA model (highlighted with gray background in this figure) did not reach statistically significant differences when explored using univariate statistical methods. In contrast, IL-1α was significantly higher in PHZ at 20–26 h after surgery in univariate analysis but did not contribute to the multivariate model. Median (line) and individual values presented: *p < 0.05 (Wilcoxon signed-rank test). IFN= interferon, IL= interleukin, IP-10= interferon-gamma induced protein 10, LPR= lactate pyruvate ratio, MCP= monocyte chemoattractant protein, MDC= macrophage derived chemokine, MIP= macrophage inflammatory protein, OPLS-DA= orthogonal projection to latent structures discriminant analysis, TARC= thymus and activation regulated chemokine, TNF= tumor necrosis factor, VEGF-A= vascular endothelial growth factor A

Univariate analysis of cytokines and chemokines revealed a significantly higher level of IL-2, IL-8, and IL-1α at 20–26 h post surgery, whereas IL-6 and IL-4 levels were increased at 44–50 h in the PHZ when compared with the SNX (Fig. 3; *p* < 0.05). Several cytokines, including most anti-inflammatory, displayed a similar pattern in the PHZ and the SNX (see Supplemental digital content Fig. 1). Chemokines had, in general, lower levels in the PHZ than in the SNX; however, only thymus and activation regulated chemokine levels were statistically different (see Supplemental digital content Fig. 2).

### Multivariate Analysis

**Fig. 4:**
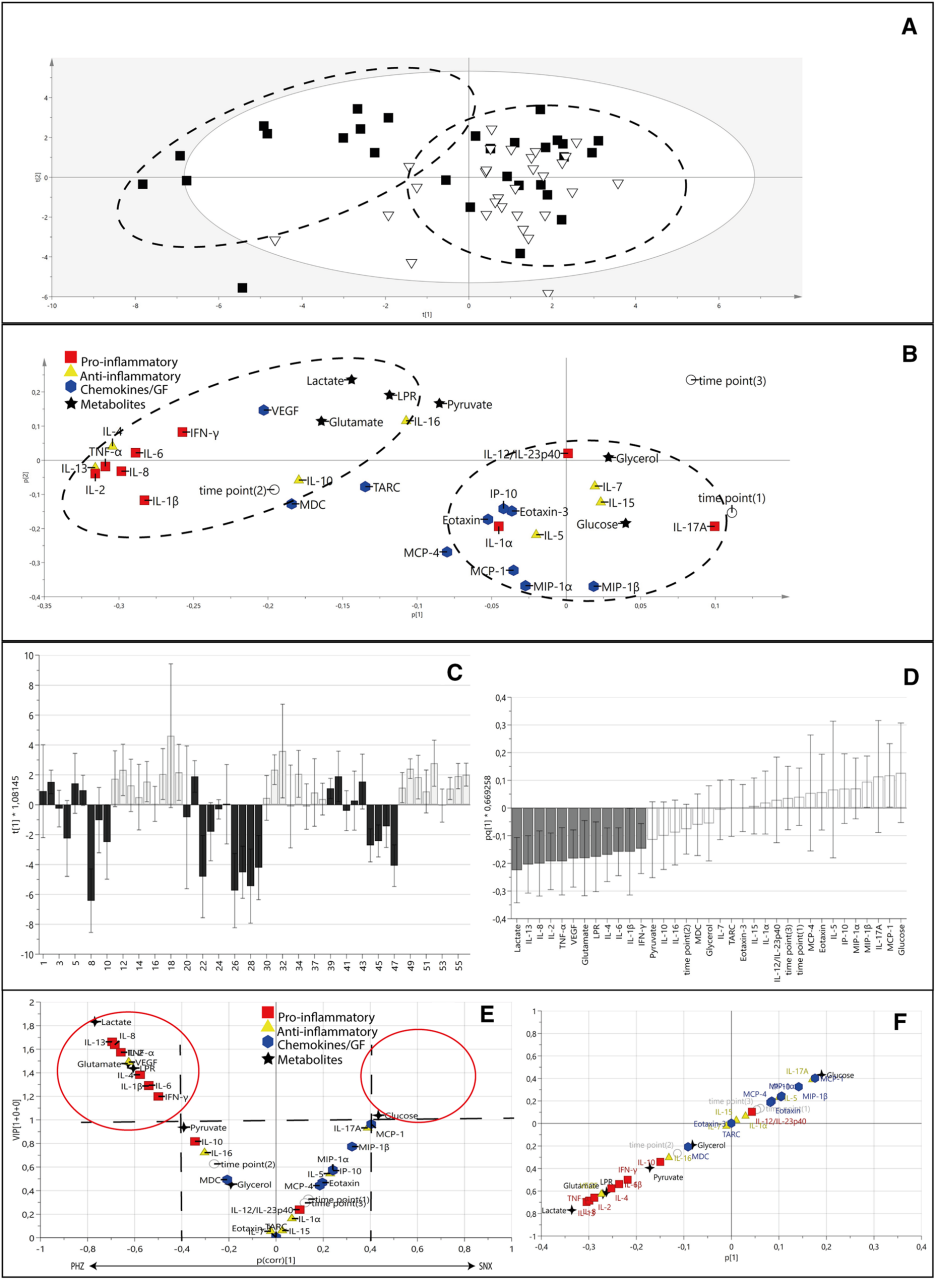
Multivariate data analysis showed separation between PHZ and SNX. **a,** Principal component analysis (PCA) score scatter plot of cytokine and metabolite data from PHZ (black squares) and SNX (white inverted triangles) show a separation between the two groups. The PHZ data points gravitate toward the upper left quadrant, whereas the SNX data points predominantly cluster toward the lower right quadrant (dashed black circles). **b** Corresponding PCA loading scatter plot shows clustering of proinflammatory cytokines (red squares) and metabolites associated with a deranged metabolic state (black stars) to the left upper quadrant corresponding to the predominant location of PHZ data (black dashed circle) in part **a**. In contrast, MD-glucose and MD-glycerol levels cluster with SNX data points, as do chemokines (blue hexagons) and anti-inflammatory cytokines (yellow triangles). Time point 2 (20–26 h after surgery; unfilled circle) correlates with higher proinflammatory cytokine expression compared with time points 1 (4–10 h after surgery) and 3 (44–50 h after surgery), respectively, apart from IL-17A, which is distinctly correlated with time point 1. **c** Score plot of OPLS-DA model (one component) showing model separation of PHZ (black columns) and SNX (white columns) data points. **d** Loading plot of OPLS-DA model showing which variables contribute strongest (gray bars) to group separation within the model, with 95% confidence interval (error bars). **e** Volcano plot for the OPLS-DA model shows variables more specific to PHZ to the left on the x-axis and those associated with SNX to the right. Variables that contribute the strongest to group discrimination (i.e., with a |*p*(corr)|> 0.4 and VIP > 1) are placed within the red circles. Lactate, pyruvate, LPR, and glutamate (black); proinflammatory cytokines IL-8, TNF-α, IL-2, IL-1β, IL-6, and IFN-γ (red); anti-inflammatory cytokines IL-13 and IL-4 (yellow); and growth factor VEGF-A (blue) were higher in PHZ compared with SNX. The majority of the anti-inflammatory cytokines and chemokines did not contribute significantly to the model, suggesting that their concentrations were similar in the PHZ and the SNX. **f** S-plot indicating strength of contribution of each variable to the OPLS-DA model. GF= growth factor, IFN= interferon, IL= interleukin, IP-10= interferon-gamma induced protein 10, LPR= lactate pyruvate ratio, MCP= monocyte chemoattractant protein, MDC= macrophage derived chemokine, MIP= macrophage inflammatory protein, OPLS-DA= orthogonal projection to latent structures discriminant analysis, PHZ= perihemorrhagic zone, SNX= seemingly normal cortex, TARC= thymus and activation regulated chemokine, TNF= tumor necrosis factor, VEGF-A= vascular endothelial growth factor A, VIP = variable influence on projection.

Principal component analysis illustrated clustering in the data (Fig. 4a). Hotelling’s T2 analysis showed two critical outliers (> 99% T2) which were subsequently removed, without altering neither global nor pair wise test results. Score scatter plot for PCA showed inherent group separation in the data (Fig. 4a). The corresponding loading scatter plot showed PHZ data clustering largely due to metabolites and proinflammatory cytokines (Fig. 4b). The proinflammatory and metabolite variables clustered near time point 2 (20–26 h post surgery) as opposed to time points 1 and 3 (4–10 and 46–50 h post surgery, respectively).

**Fig. 5:**
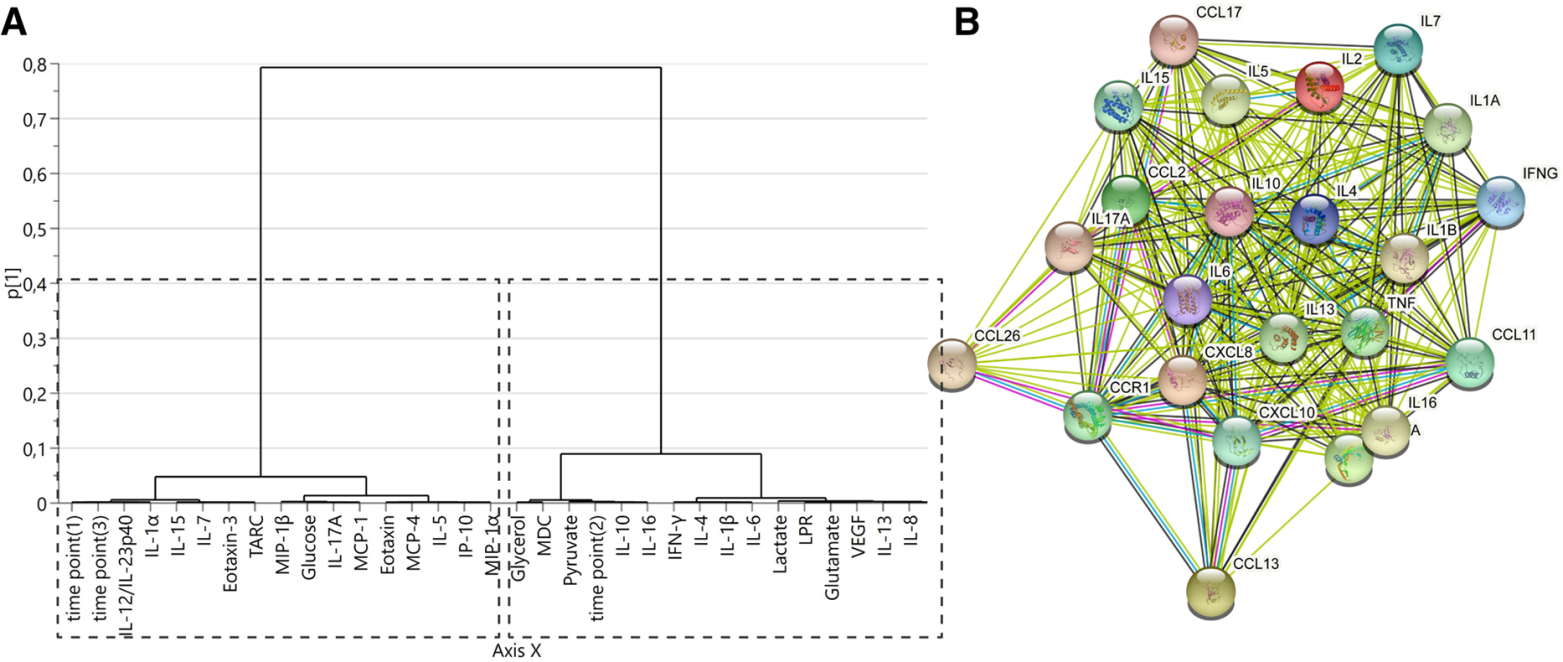
Hierarchical cluster analysis dendrogram depicting two major clusters in the data and protein-protein interaction (PPI) network analysis. **a** Hierarchical cluster analysis revealed two major clusters in the metabolic markers and inflammatory mediators of the OPLS-DA model. **b** The STRING PPI network analysis of the inflammatory mediators reveals several connections. CCL= C–C motif chemokine ligand, CCR= C–C motif chemokine receptor, CXCL= C-X-X motif chemokine ligand, IL= interleukin, OPLS-DA= orthogonal projection to latent structures discriminant analysis, PHZ= perihemorrhagic zone, SNX= seemingly normal cortex, TNF= tumor necrosis factor

A supervised OPLS-DA model was then fitted (OPLS-DA [1 + 0 + 0] *R*^2^ = 0.179, *Q*^2^ = 0.23; *p (CV-ANOVA)* < 0.001) with MD catheter location as class (Fig. 4c). Four metabolites and eight cytokines showed the strongest contribution to the difference between the PHZ and SNX (|*p*(corr)|> 0.4 and VIP > 1; Fig. 4e, Table 2). Notably, low-molecular weight metabolites suggestive of a metabolic crisis and tissue injury such as lactate, LPR, pyruvate and glutamate, were significantly higher in PHZ when compared to the SNX. Furthermore, the proinflammatory cytokines TNF-α, IL-6, IFN-γ, IL-1β, IL-8, and IL-2 were higher in PHZ compared to SNX, as were the anti-inflammatory cytokines IL-13 and IL-4, and growth factor VEGF-A. A hierarchical cluster analysis dendrogram of the OPLS-DA data demonstrated that inflammatory mediators and metabolites clustered into two major groups (Fig. 5a). Network analysis showed highly connected inflammatory mediators (Fig. 5b).

**Table 1:** Patient characteristics

Patient #	Sex	Age (yrs)	Time to surgery (h)	Sampling time (h)	Dist-PHZ (mm)	Dist-SNX (mm)	Volume (mL)	ICH side	ICH location	Outcome (mRS)
1	M	51	10	112	0	27	89	L	BG	LTF
2	M	71	14	74	6	38	93	R	LO	2
3	M	64	82	148	6	20	43	R	BG	4
4	F	70	6	58	4	22	89	L	LO	2
5	M	68	6	130	2	34	114	R	BG	4
6	M	64	6	86	8	30	45	L	BG	1
7	M	63	58	146	9	11	44	L	BG	3
8	M	69	20	50	6	19	78	R	LO	6
9	F	64	12	90	0	27	33	L	BG	2
10	M	57	64	56	18	19	76	L	BG	3

BG = basal ganglia; Dist-PHZ = distance from PHZ catheter to ICH; Dist-SNX = distance from SNX catheter to ICH; F = female; h = hours; ICH = intracerebral hemorrhage; L = left; LO = lobar; LTF = lost to follow up; M = male; mRS = modified Rankin Scale; Patient # = patient number; R = right; yrs = years

**Table 2:** Variables contributing strongly to OPLS-DA model discriminating between PHZ and SNX

Variable	❙*p*(corr)❙	VIP
Lactate	− 0.77687	1.82257
IL-13	− 0.70948	1.66652
IL-8	− 0.6973	1.63755
Glutamate	− 0.68288	1.61133
TNF-α	− 0.66947	1.57238
IL-2	− 0.66011	1.55044
LPR	− 0.65954	1.52502
VEGF-A	− 0.63103	1.48236
IL-4	− 0.58531	1.37511
IL-1β	− 0.55034	1.29242
IL-6	− 0.54935	1.29079
IFN-γ	− 0.50683	1.19044
Pyruvate	− 0.46304	1.07022

VIP > 1 and absolute *p*(corr) > 0.4 are used to determine which variables are most important for the model, [43] meaning these variables contribute most to model group separation

IFN= interferon, IL= interleukin, LPR= lactate-pyruvate ratio, OPLS-DA= orthogonal projection to latent structures discriminant analysis, PHZ= perihemorrhagic zone, SNX= seemingly normal cortex, TNF= tumor necrosis factor, VEGF-A= vascular endothelial growth factor A, VIP= variable influence on projection

## Discussion

By placing paired microdialysis catheters, one in the perihemorrhagic zone (PHZ) of the evacuated ICH and one in noninjured seemingly normal cortex (SNX) we could demonstrate a higher expression of predominantly proinflammatory cytokines in the PHZ compared to the SNX, most evident at 20–26 h post-surgery. Proinflammatory cytokines IL-8, TNF-α, IL-2, IL-1β, IL-6, and IFN-γ, were significantly higher in PHZ compared to SNX.

Analysis of low-molecular weight metabolites showed a persistent metabolic crisis in the perihemorrhagic tissue, consistent with our previous study [39]. The high levels of MD-glutamate in the PHZ suggests an ongoing neuronal death, and a subsequent plausible cell membrane degradation could contribute to the gradually increasing MD-glycerol levels.

Increased proinflammatory cytokine expression in perihemorrhagic tissue including expression of IL-1β, TNF-α, and MIP-1α has previously been observed in animal models [18, 44–47], however, there are no previous studies of cytokine expression in brain tissue of patients with ICH. Our present findings support the presence of a proinflammatory environment in the tissue surrounding an ICH and provides information on the temporal profile of the inflammatory cascades. VEGF-A, which mediates increased permeability of the blood–brain barrier [48, 49], was distinctly higher in PHZ compared with SNX, which may then potentially aggravate vasogenic edema and cause further secondary brain injury.

The inflammatory response may also have a beneficial role in ICH, orchestrated by the M2 microglial phenotype, involved in clearing debris and tissue repair following ICH [13–16, 18–21, 50–56]. IL-13 and IL-4, increased in the PHZ in the present study, are known to be anti-inflammatory or have a modulating effect on proinflammatory mediators [57]. IL-4 has been shown to convert M1 phenotype microglia toward an M2 phenotype [58, 59], further illustrating that tissue reaction to ICH is complex and the balance of anti- and proinflammatory mediators may shift over time.

A previous study of patients with TBI showed the recovery of 42 cytokines from interstitial fluid, and demonstrated distinct temporal profiles in 16 of these. Similarly to the present study, TNF-α, IL-8, IL-1β, and IL-6 were found to peak within the first two days of monitoring [32]. Although TBI and ICH may share some characteristics, they are also two distinct disease entities with unique pathophysiologies.

Cytokines can have both a dual and conflicting role. To classify them as either anti- or proinflammatory is overly simplistic, as the inflammatory mediators constitue an intricate network of paracrine and autocrine molecules whith both positive and negative feedback loops [60]. Therefore, it is important to consider both the context, the timing and the target of any given cytokine in order to understand and interpret its action as either a driver or a moderator of the immune response. In particular IL-6, TGF-β and IFN-γ can be either proinflammatory or anti-inflammatory, depending on timing and target [61]. In similarity to our present study, both anti-inflammatory and proinflammatory cytokines are released simultaneously in the tissue following TBI [62, 63].

## Limitations

A limitation of cerebral microdialysis is the lack of control levels, and interpretation of absolute levels must be made with caution. Our approach enables evaluation of the temporal profile of the inflammatory cascades but also a comparison with a relatively uninjured brain region, the SNX, as it is exposed, but not injured, by the surgical approach. Because the MD catheters are placed by free-hand, some variability of their placement is inevitable. Although this variation was small and within reasonable limits (typically < 10 mm) we cannot exclude that this variability may have influenced the results. Routine CT scans were performed postoperatively in all patients when clinically indicated, however, not at the same time points as inflammatory mediators were analyzed. Furthermore, these bedside CT scans used in our neurocritical care unit were performed using thick sections. For this reason, perihemorrhagic and postsurgical edema and its potential correlation to inflammatory mediators could not be assessed.

None of the patients had convulsive seizures, nor were any nonconvulsive seizures detected using scalp electrode electroencephalogram (EEG). However, we did not have the possibility to monitor the perihemorrhagic tissue by stereotaxically inserted deep EEG electrodes. Any local seizure activity would influence tissue metabolism and homeostasis locally. This could be explored by deep, local tissue EEG monitoring in a future study.

Relative recovery [26, 64] was not determined in the present study, however, it can be assumed to be similar between the paired catheters, thus differences between these should not be affected by changes in recovery.

This study is limited by the small number of patients, explained by the highly complex setup and the fact that only 3% of all patients with ICH in Sweden undergo surgical evacuation [65], which limits the generalizability of the results.

Ethical considerations preclude the insertion of microdialysis catheters in patients who do not undergo surgical ICH evacuation, therefore control patients are not available. The surgical approach, although performed using microsurgical technique to minimize the trauma to the surrounding tissue, may have contributed to the release of inflammatory mediators. However, the distinct differences between the two catheters argues against a marked tissue reaction and cytokine release from insertion of the microdialysis catheter per se.

The study design precludes the discovery of cytokine peaks later than 50 h post surgery, as has previously been demonstrated for IL-12p70 and IL-10 in patients with TBI [32]. Furthermore, any peak prior to MD insertion would also evade detection. In addition, the variable time to surgery from ICH onset may have introduced uncontrolled data variability, as the ICH-induced inflammatory response likely was in different stages when surgery was performed and when MD-sampling was initiated. Nevertheless, our results show that the most significant changes occurred already at 20–26 h after surgery suggesting an early inflammatory peak following ICH.

In this present study the OPLS-DA model was employed to discern overall correlations and differences in the data. A highly significant model was developed, but the *R*^2^ and *Q*^2^ point to a rather large amount of noise in the data. The small sample size also precludes having separate training sets and validation data sets for the multivariate models. The model is robust enough for exploratory analysis of overall correlation patterns in the data. Further studies are required to verify the findings in this present study.

## Conclusions

In this study, we compared levels of inflammatory mediators in the interstitial fluid of the PHZ with that of the CNX following surgical evacuation of ICH. We found an increased expression of proinflammatory cytokines in the PHZ 20–26 h after surgery. This acute inflammatory response may constitute a target for future therapies aiming to reduce secondary brain injury.

## Supplementary Information

Below is the link to the electronic supplementary material.

Supplementary file1 (PDF 76 kb)

Supplementary file2 (PDF 280 kb)
