# Supplementary material for: Intracerebral Proinflammatory Cytokine Increase in Surgically Evacuated Intracerebral Hemorrhage: A Microdialysis Study
Source: Neurocrit Care. 2021 Nov 30;36(3):876–87. doi: 10.1007/s12028-021-01389-9 (PMC9110446; doi:10.1007/s12028-021-01389-9)
Supplement: Supplementary file 1 — Supplementary file1 (PDF 76 kb) [file 12028_2021_1389_MOESM1_ESM.pdf]

Supplemental Digital Content 1

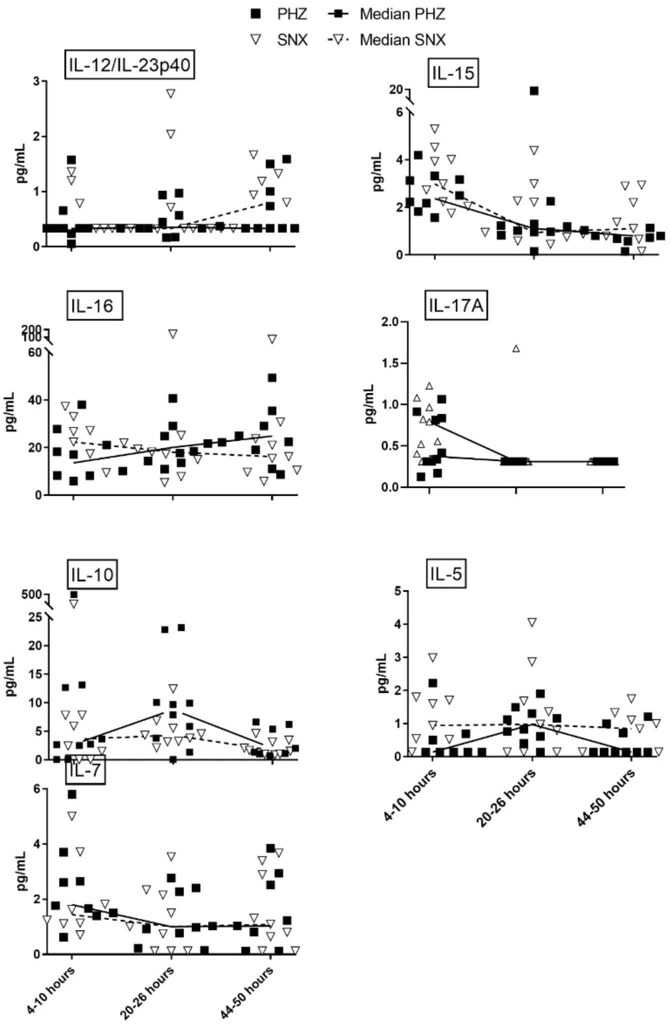

Supplemental Digital Content 1, Figure. Univariate analysis of cytokines (median and individual values; * = $p < 0.05$) which did not differ significantly between perihemorrhagic zone (PHZ) and seemingly normal cortex (SNX).

Univariate analysis of the concentration of these cytokines found no significant difference between perihemorrhagic zone (PHZ) and seemingly normal cortex (SNX). This was corroborated also by the multivariate data analysis, apart from for VEGF-A which was elevated in the PHZ and contributed to distinction between PHZ and SNX in the supervised OPLS-DA model (figure 4C in main manuscript).

Abbreviations: PHZ = perihemorrhagic zone; SNX = seemingly normal cortex; IL = interleukin; TNF = tumor necrosis factor; VEGF-A= vascular endothelial growth factor A; LPR = lactate pyruvate ratio; IFN = interferon; MDC = macrophage derived chemokine; TARC = thymus and activation regulated chemokine; MCP = monocyte chemoattractant protein; IP-10 = interferon-gamma induced protein 10; MIP = macrophage inflammatory protein.
