# Supplementary material for: Intracerebral Proinflammatory Cytokine Increase in Surgically Evacuated Intracerebral Hemorrhage: A Microdialysis Study
Source: Neurocrit Care. 2021 Nov 30;36(3):876–87. doi: 10.1007/s12028-021-01389-9 (PMC9110446; doi:10.1007/s12028-021-01389-9)
Supplement: Supplementary file 2 — Supplementary file2 (PDF 280 kb) [file 12028_2021_1389_MOESM2_ESM.pdf]

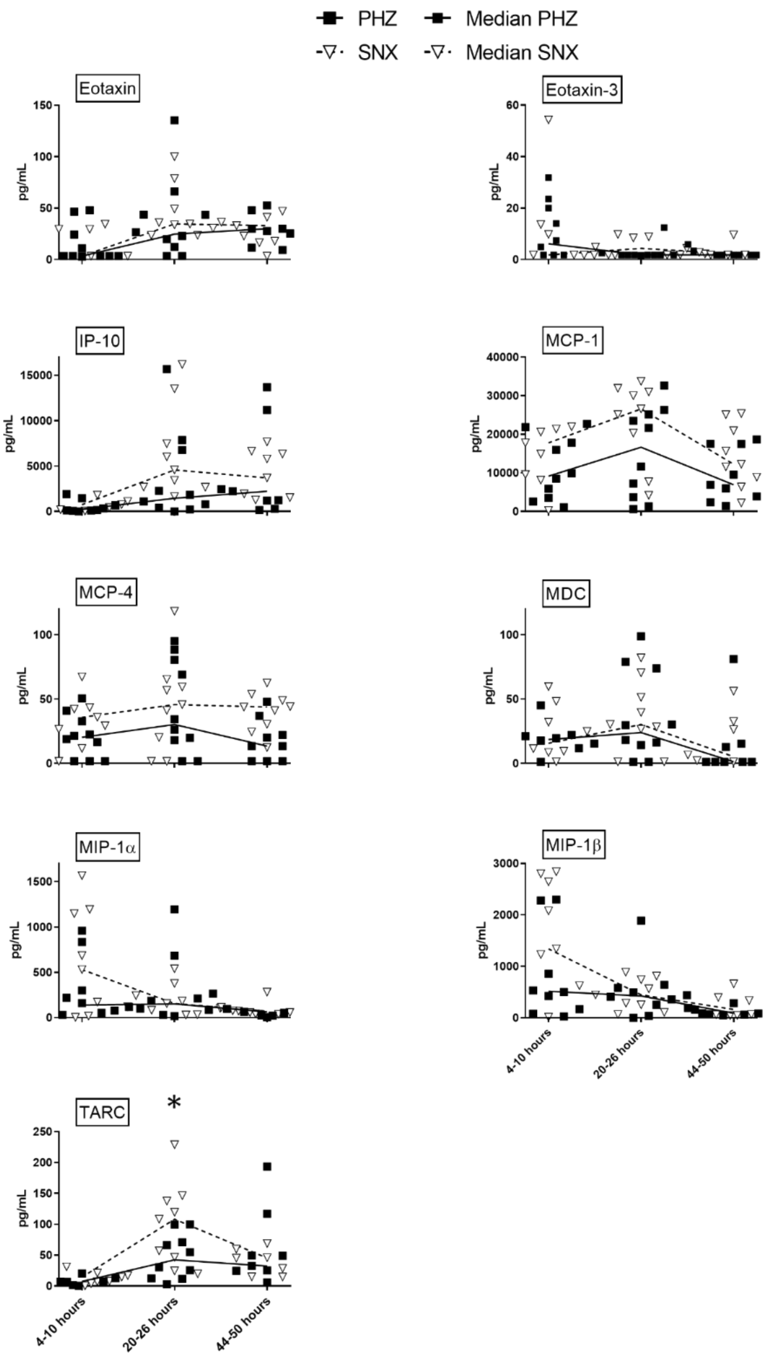

Supplemental digital content 2; Figure. A significantly higher expression of TARC in seemingly normal tissue (SNX) compared to perihemorrhagic zone (PHZ) was found using univariate analysis at 20-26 hours after surgery (median and individual values; * = $p < 0.05$). Furthermore, chemokines tended to show higher concentrations in the SNX than in the PHZ although without reaching statistical significance.

Abbreviations: = interleukin; TNF = tumor necrosis factor; VEGF-A= vascular endothelial growth factor A; LPR = lactate pyruvate ratio; IFN = interferon; MDC = macrophage derived chemokine; TARC = thymus and activation regulated chemokine; MCP = monocyte chemoattractant protein; IP-10 = interferon-gamma induced protein 10; MIP = macrophage inflammatory protein.
